# Central nervous system and muscular bundles preserved in a 240 million year old giant bristletail (Archaeognatha: Machilidae)

**DOI:** 10.1038/srep46016

**Published:** 2017-04-07

**Authors:** Matteo Montagna, Joachim T. Haug, Laura Strada, Carolin Haug, Markus Felber, Andrea Tintori

**Affiliations:** 1 Dipartimento di Scienze Agrarie e Ambientali - Università degli Studi di Milano, Via Celoria 2, I-20133 Milano, Italy; 2 Functional Morphology, Department of Biology II and GeoBio-Center, LMU Munich, Großhaderner Str. 2, 82152 Planegg-Martinsried, Germany; 3 Dipartimento di Scienze della Terra “Ardito Desio” - Università degli Studi di Milano, Via Mangiagalli 34, I-20133 Milano, Italy; 4 Consulenze Geologiche e Ambientali SA, Via Comacini 31, CH-6834 Morbio Inferiore, Switzerland

## Abstract

Among the incomparably diverse group of insects no cases of central nervous system (CNS) preservation have been so far described in compression fossils. A third of the fossil insects collected from a 240–239 million year old (Ma) level at Monte San Giorgio UNESCO World Heritage (Switzerland-Italy) underwent phosphatization, resulting in the extraordinary preservation of soft tissues. Here we describe *Gigamachilis triassicus* gen. et sp. nov. (Archaeognatha: Machiloidea: Machilidae) that, with an estimated total length of ~80 millimeters, represents the largest apterygote insect ever recorded. The holotype preserves: (*i*) components of the CNS represented by four abdominal ganglia, optic lobes with neuropils and compound retina; (*ii*) muscular bundles. Moreover, *G. triassicus*, possessing morphological features that prompt its assignment to the extant archaeognathan ingroup Machilidae, places the origin of modern lineages to Middle Triassic. Interestingly, at Monte San Giorgio, in the same stratigraphic unit the modern morphology of *G. triassicus* co-occurs with the ancient one represented by *Dasyleptus triassicus* (Archaeognatha: †Monura). Comparing these two types of body organization we provide a new reconstruction of the possible character evolution leading towards modern archaeognathan forms, suggesting the acquisition of novel features in a lineage of apterygote insects during the Permian or the Lower Triassic.

The exceptional preservation of soft tissues in compression fossils has been reported only in few occurrences within invertebrates, as in the case of Cambrian arthropods from Chengjiang (e.g., refs 1, 2, 3, 4, 5) and Burgess Shale (e.g., refs 6, 7, 8). Such soft tissue preservation has been only exceptionally achieved by tissue mineralization, usually involving pyritisation and phosphatization^9^,^10^ or, in the case of non-mineralized fossils, in the form of kerogenized carbon films^11^. Phosphatization of organic matter is a process occurring in anoxic conditions and it is usually mediated by bacteria^9^; the diffusion of phosphate released from the decaying animal’s tissues to the surrounding media is prevented by a microbial film acting as insulation^10^. Approximately one third of the fossil insects collected from the Kalkschieferzone (239.51 ± 0.15 Ma)^12^ of Monte San Giorgio (UNESCO World Heritage Site, Switzerland-Italy) are completely or partially phosphatized^13^. In this Lagerstätte, phosphatization has been observed also in crustaceans but, interestingly, never among vertebrates (A.T. pers. obs). Here we describe two completely phosphatized specimens we assign to an extant bristletail group (Insecta: Archaeognatha: Machiloidea: Machilidae). They exhibit giant size, compared to known extinct and extant species (overall organism length of ~80 mm, body plus filum terminale), and extraordinarily preserved internal soft tissues, notably components of the central nervous system (CNS) and muscular bundles.

The fossil record of Archaeognatha (Machiloidea plus †Monura) is sparse and is often represented by fragmentary material. Specimens attributed to archaeognathan lineages span from Late Devonian (~379 Ma)^14^ to Miocene (~13 Ma)^15^. So far, most of the Paleozoic and Mesozoic samples are representatives of *Dasyleptus*, the only ingroup of †Dasyleptidae and †Monura (hence equivalent to these), while most of Cenozoic species are representatives of *Machilis* (Machilidae). The oldest bristletail fossils are fragments that date back to the Devonian Period^14^,^16^. A specimen described from Gaspé Bay (390–392 Ma) is a head capsule plus a separate thoracic fragment from the same organism^16^. The presence of large but dorsally not converging eyes on the head capsule, a synapomorphic trait of all modern bristletails^17^, suggest the assignment of this specimen to the Paleozoic monuran rather than to modern lineages. Findings from the compressed shales of Gilboa (376–379 Ma) are represented by partial tergites plus an eye fragment. The tergites bear coffin-shaped sockets compatible with structures present in extant bristletails, while the eye fragment was “*tentatively identified as belonging to machilid insect*” by the authors^14^. So far, fossils of certain attribution to Machilidae are known only from the Eocene^18^,^19^,^20^. Complete or almost complete Palaeozoic specimens of clear systematic affiliation have been described only for the extinct genus *Dasyleptus* (†Monura)^21^,^22^,^23^,^24^,^25^,^26^,^27^. Three specimens of *Dasyleptus triassicus* (†Monura) have been recovered from the same stratigraphic unit of our findings^28^, and many specimens from the German Upper Buntsandstein deposits (Obere Röttonsteine, Early Anisian) in Lower Franconia and Thuringia^29^. These findings extend the presence of *Dasyleptus* well after the end-Permian mass extinction (252.3 Ma) and demonstrate that these organisms were still quite common in the Middle Triassic. Here we provide an updated reconstruction of character evolution leading towards the modern forms of bristletails based on the comparison between the ancient-type *D. triassicus* and the modern-type represented by the new species described. Furthermore, we provide evidence for the acquisition of a new body organization in a lineage of apterygote insects at the end of the Permian or during the Triassic Period, after the end-Permian mass extinction.

## Results

### Systematic palaeontology

Euarthropoda sensu Walossek, 1999^30^; Insecta Linnaeus, 1758; Archaeognatha Börner, 1904; Machiloidea Handlirsch, 1904; Machilidae Grassi, 1888; *Gigamachilis* gen. nov. http://zoobank.org/urn:lsid:zoobank.org:act:58CF94C0-30E8-4102-B4CD-918FDE929C02

### Type species

*Gigamachilis triassicus* new species here designated. http://zoobank.org/urn:lsid:zoobank.org:act:760D7E33-357C-430E-BB93-F71EF36B32DA

### Etymology

*Giga*- (from Greek gígas) means giant, referring to the very large size; -*machilis* from Machilidae to which *Gigamachilis* is ascribed; *triassicus* (Latin) refers to the Triassic Period.

### Material

The two *G. triassicus* types were recovered at the UNESCO World Heritage Middle Triassic site of Monte San Giorgio (Switzerland) in locality D (Val Mara, Meride) on the uppermost part of the Lower Kalkschieferzone. Detailed information regarding geology, dating of the collecting site and on the fossil assemblage is reported in Supplementary Note 1.

**Figure 1:**
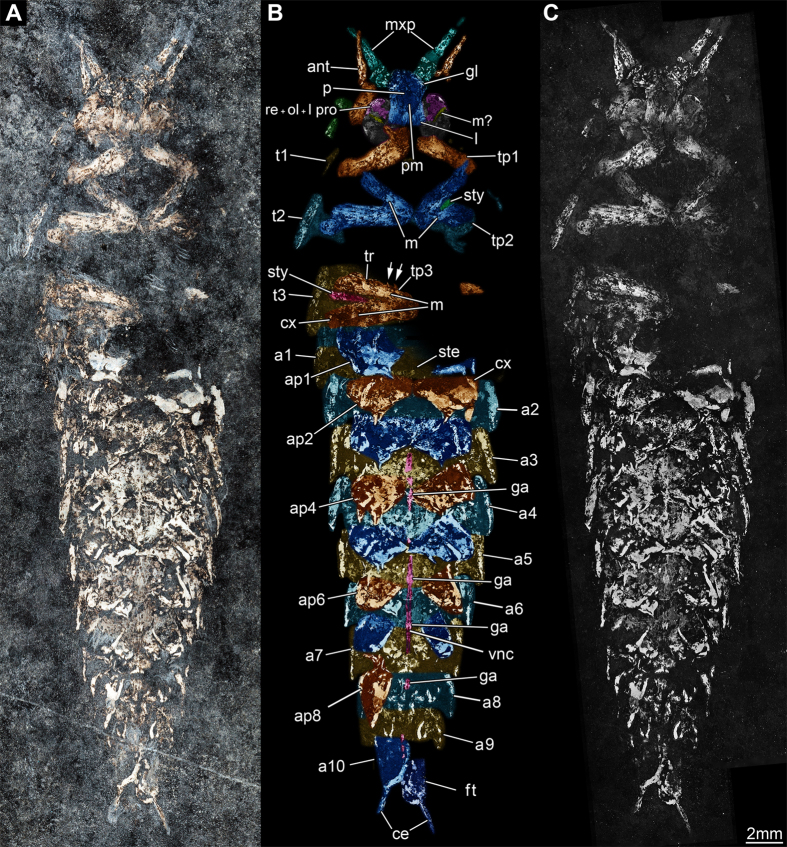
*Gigamachilis triassicus* holotype. (**A**) Macrophotography under cross-polarized light. Autofluorescence (473 nm, GFP) composite image, (**B**) color-marked version and (**C**) original image. Abbreviations: a = abdominal segment; ant = antenna; ap = abdominal appendage; ce = cerci; cx = coxa/coxopodite; ft = filum terminale; ga = ganglion; gl = glossa; l = labium; l pro = lateral protocerebrum; m = muscle; m? = possible muscle; mxp = maxillary palp; ol = optic lobes; p = prementum; pm = postmentum; re = compound retina; ste = sternite; sty = stylus; t = thoracic segment; tp = thoracic appendage; tr = trochanter; vnc = ventral nerve cord. Arrows pointing to spines.

**Figure 2:**
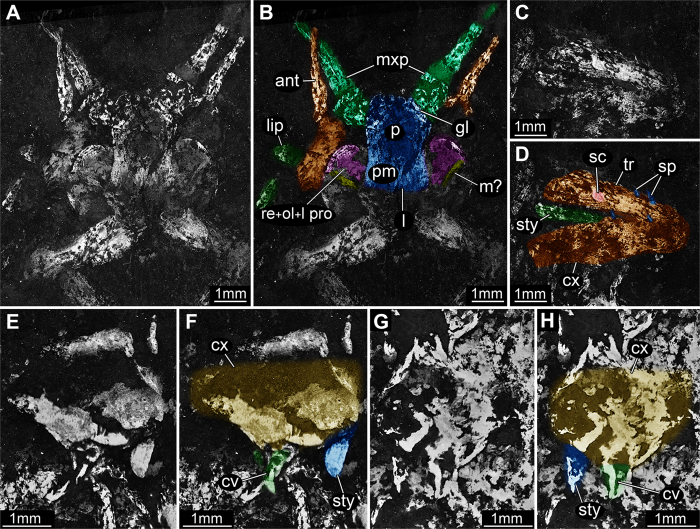
Exomorphological details of *Gigamachilis triassicus.* Head region, original image (**A**) and color-marked version (**B**). Third thoracic appendage, original image (**C**) and color-marked version (**D**). Second abdominal appendage, original image (**E**) and color-marked version (**F**). Fourth abdominal appendage, original image (**G**) and color-marked version (**H**). All composite autofluorescence images. Abbreviations as in Fig. 1 with the addition of: cv = coxal vesicle; lip = labial palp; sc = scale; sp = spines.

**Figure 3:**
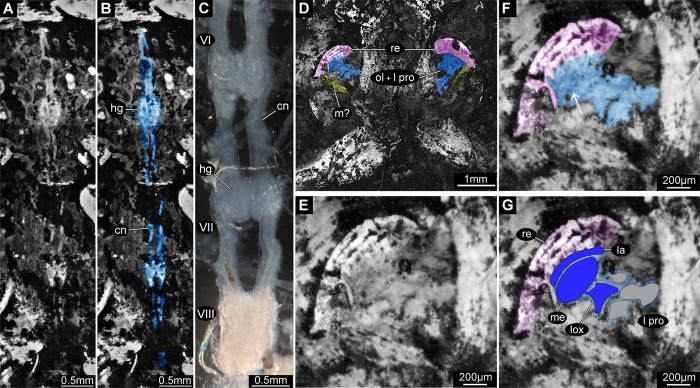
Details of *Gigamachilis triassicus* CNS. (**A**) Close-up on medio-ventral region of abdominal segments 6–8 and color-marked version (**B**); in blue, structures of ventral nerve cord, including ganglia with hemiganglia and paired connectives. (**C**) Abdominal ganglia, VI to VIII, of *Machilis* sp. ventral nerve cord, for structural comparison. (**D**) Head region highlighting the compound retina (marked purple), the optic lobes and the lateral protocerebrum (CNS structures, marked blue) and the bundle-like features interpreted as possible muscles (marked yellow). Close-up on the right compound retina, optic lobes and components of the lateral protocerebrum (**E**) and color-marked version (**F**) with arrow pointing to possible Cuccati’s bundle. Colors as in (**D**). (**G**) The same region as (**E**) and (**F**) with schematic representation of the three nested neuropils within the optic lobe (marked blue) and components of lateral protocerebrum (marked light grey). All but (**C**) composite autofluorescence; (**C**) macro-photography under transmitted light. Abbreviations: cn = connective; hg = hemiganglion = la = lamina; lox = lobula complex; me = medulla [insect brain nomenclature as in Ito *et al*.^46^].

Specimen will be deposited at Museo Cantonale di Storia Naturale di Lugano (MCSN) – Switzerland. MCSN8463 (holotype) is an almost complete specimen (Figs 1, 2 and 3) while MCSN8466 (paratype) preserves only the abdomen and the metathorax (Supplementary Fig. S1).

### Taphonomy and preservation

Holotype and paratype are fully phosphatized. The holotype preserves the entire body, including soft tissues, with the exception of the distal part of the body appendages as the maxillary palps, the antennae, the walking legs and the filum terminale. This preservation, including the loss of the delicate appendages, suggests that *G. triassicus* was rapidly transported from its original habitat to the depositional basin by a high-energy event, such as floods caused by heavy rains. The rapid transportation of the specimens to the anoxic condition of the depositional basin represents a requirement to obtain soft tissue preservation through the bacteria-mediated process of phosphatization. Since the body outline of both specimens is preserved, we can infer that underwater currents and bioturbation were absent in the depositional environment.

### Diagnosis

Huge machilids, almost twice the size of the largest species of Machilidae known so far. The pattern of coxal vesicles distribution is not congruent with any previously described form, both extinct and extant.

### Description

*G. triassicus* is ascribed to Archaeognatha based upon the following characters: large maxillary palps with several elements, abdominal coxopodites with coxopodal vesicles and styli, paired annulated cerci and filum terminale (basal parts preserved). The presence of styli-like structures on the second thoracic leg and of scales on appendages prompts its attribution to the extant group Machilidae.

Here we describe the new taxon based on the almost complete holotype (MCSN8463; Figs 1, 2 and 3); the description of the partially preserved paratype (Supplementary Fig. S1) is provided in the Supplementary Note 1.

General habitus: specimen with head and thorax slightly rotated in the sagittal plane, only visible in ventral view; body length from the apex of the head to the apex of the last abdominal segment, thus excluding filum terminale, of 40 mm; body maximum width of 12.5 mm (second thoracic segment) (Fig. 1). On the base of the ratio between the length of the filum terminale and that of the whole organism in extant taxa, the length of *G. triassicus* was estimated in approximately 80 mm.

Head: eyes very large, developed laterally. Antennae partially preserved, only proximal parts visible: antennal socket, scapus, pedicellus and a portion of the annulated flagella (length 2.9 mm). Mouthparts partially preserved. The terminal element of the right labial palp and the first three elements of the large leg-like maxillary palps are visible; labium prementum, maxillary palpifers and glossae are partially visible.

Thorax: total length 9.8 mm, maximum width at mesothorax 12.5 mm. Impression of lateral rims of pronotum and mesonotum preserved on the right side (respectively 1.8 and of 3.6 mm long; mesonotum thickness 0.6 mm), rim of mesonotum partially preserved on the left side. Procoxae (length: right 3.9 mm, left 3.3 mm), proximal part of protrochanters, mesocoxae (length: right 4.2 mm, left 2.8 mm) and mesotrochanters (length: right 3.5 mm, left 4.3 mm) preserved. Left mesocoxa bearing the proximal part of the coxal stylet (length 0.9 mm). Trochanter distally lobe-shaped. Right metacoxa (length 4.7 mm) and metatrochanter preserved (length 9.2 mm), the first bearing coxal stylet (length 4.3 mm), setae (length 0.35 mm) and scales (Figs 1 and 2). Left metatrochanter only partially visible.

Abdomen: composed of 10 visible segments, the first only partially visible on the right side, the last segment bearing the proximal part of the two cerci and of the filum terminale. Total length 26.3 mm, maximum width at abdominal metamere I 10.1 mm. Inferior rim of the tergite and right coxopodite preserved on abdominal metameres I to VIII, whereas in metamere IX these structures are visible but poorly preserved (Fig. 1). Coxopodal vesicles present on abdominal segments I to VII (Figs 1 and 2E–H). Abdominal styli are clearly visible on abdominal appendages II (left) and IV (right). Cerci and filum terminale on segment X partially preserved.

### Soft tissue preservation

Notably, in the holotype of *Gigamachilis triassicus* soft tissues are preserved, namely parts of the central nervous system and muscular bundles within legs, abdominal appendages and in the head. The following structures of the central nervous system, are preserved: (*i*) optic lobes and, possibly, components of the lateral protocerebrum (right side) (Figs 1, 2A,B and 3D–G); (*ii*) partial ventral nerve cord composed of four pairs of abdominal ganglia with their connectives (Figs 1 and 3A,B). Symmetrically to the postmentum, two semispherical structures are preserved (Figs 1, 2A,B and 3D–G). Due to their position and to the striated structures they are interpreted as compound retinae (Fig. 3D–G). Posteriorly to the retina the optic lobes are visible (Figs 1, 2A,B, 3D–G). On the right side the outline of the three nested retinotopic neuropils characteristic of the optic lobes of extant archaeognathans (Fig. 3E–G) can be distinguished, namely, from outside to inside: the lamina, the medulla on which it is possible to recognize the Cuccati’s bundle (indicated by the arrow in Fig. 3F) and the protolobula. In addition, three other areas, possibly belonging to the lateral protocerebrum are preserved (Fig. 3D–G). A bundle-like feature is visible below the optic lobes; considering its position and its fibrous nature, it might represent segmental cephalic muscular bundles such as those present below the posterior tentorium or as the superimposed muscles of the labial palp (distal part of the labial right palp visible in Fig. 2A,B).

More clearly than in the head region, in four abdominal segments of *G. triassicus* ganglia joined by their paired connectives are visible (Figs 1 and 3A,B). The exceptional preservation of these structures allows the identification of two hemiganglia in three out of the four preserved ones and, possibly, the commissure in ganglion VIIa and VIIIa. They are compatible with neuropils within the ganglia (length and width of the ganglia: VIa ~440 μm, ~320 μm; VIIa ~580 μm, ~310 μm; VIIIa ~370, ~260 μm).

Muscular bundles, hypothesized as femur-trochanter and adductor muscles are preserved respectively in the mesotrochanter and within the right hind leg in coxa and trochanter (Figs 1B,C and 2C,D). In addition, within abdominal plates I to IV muscles of stylets and of coxal vesicles are visible.

## Discussion

*Gigamachilis triassicus*, with an estimated total length of ~80 millimeters, is known from two phosphatized specimens preserved in ventral view. The exceptional preservation of soft tissues at ultrastructural level observed in *G. triassicus* includes abdominal ganglia, compound retina, optic lobes with the possible presence of the three nested neuropils found in modern archaeognathans, components of the lateral protocerebrum and muscular bundles. This preservation occurred through the microbially mediated taphonomic process of phosphatization^9^ and it has never been reported so far among compression fossils of terrestrial arthropods. A remarkable case of such exceptional preservation was previously observed in a specimen of *Mesolimulus walchi* from the Upper Jurassic, where spiral and coccoid bacteria forming a biofilm were preserved in addition to the horseshoe crab musculature^31^. In the Kalkschieferzone of Monte San Giorgio approximately one third of the insects recovered are completely or partially phosphatized^13^. Noteworthy, the phosphatized specimens belong to insect groups such as bristletails and stoneflies (larvae), in which the cross-link between proteins of the exocuticle and quinone occurs only in limited parts of the exoskeleton. In the Kalkschieferzone, phosphatization occurred also in other arthropods (i.e., crustaceans) but not in vertebrates (A.T. pers. obs.). The depositional environment of the Kalkschieferzone, a shallow lagoon adjacent to a carbonate platform^32^,^33^, has likely facilitated a rapid process of fossilization, which prevented the consumption of organic matter and allowed the preservation of soft tissues together with their fine structural features. The presence of clay-chips beds, rich in algal film fragments^32^,^33^, may be considered as a clue that in the depositional environment of the Kalkschieferzone the conditions for the microbially mediated phosphatization of organic matter were established.

Here, for the first time in compression fossils of terrestrial arthropods, components of the CNS are preserved. The ventral nerve cord exhibits a homonomous metameric pattern, as to be expected. Notably, the ganglia of ventral nerve cord observable in *G. triassicus* highly resemble those of extant Machilidae (Fig. 3C). In the optic lobes, the number and the relative position of the three nested retinotopic neuropils correspond to those of extant bristletails, indicating a phenotypic stability of these structure lasting at least ~240 My (extant archaeognathan optic lobes reported in Sinakevitch *et al*.^34^ Figure 9D); for an exemplary review on the organization of the optic lobes across crustaceans and insects see Strausfeld^35^.

**Figure 4:**
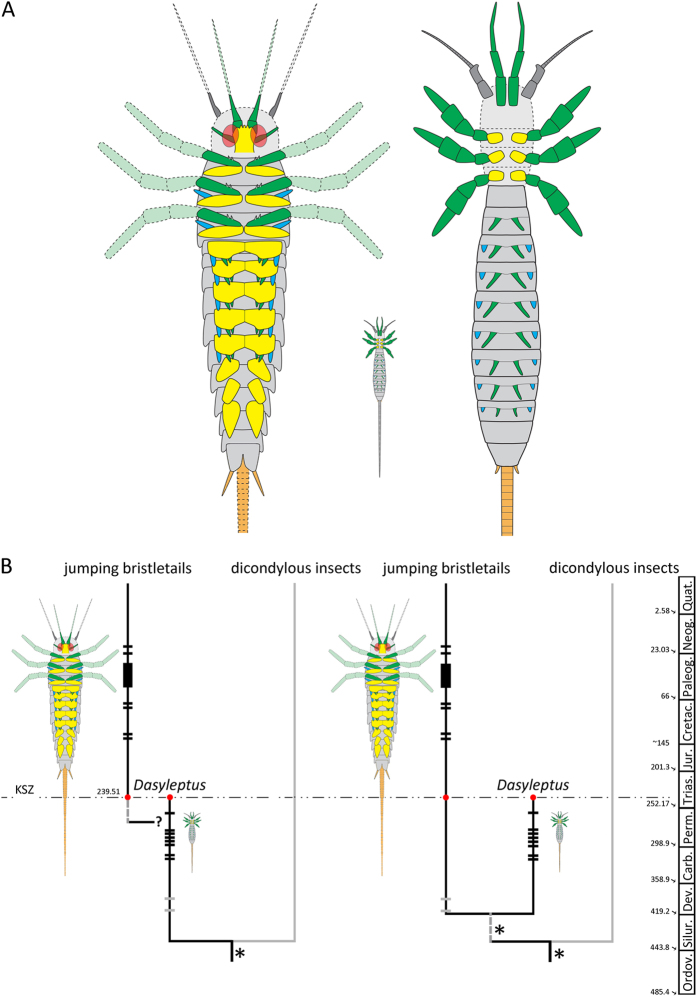
Schematic reconstructions and alternative scenarios of Archaeognatha evolution. (**A**) Reconstruction of *Gigamachilis triassicus* and *Dasyleptus triassicus* in ventral view. Coxa or coxopodite (=basipod of Euarthropoda) marked yellow; endopod and derivatives marked green; exopod derivatives in blue. Left: *G. triassicus*. Right: *D. triassicus*, based on information provided by Bechly and Stockar^28^; two pairs of ventral structures (visible in the original figures) have been reconstructed: the median one originally interpreted as the styli is here re-interpreted as eversible vesicles (due to position correlation; in green), the lateral smaller ones represents the styli (in blue). Middle: *D. triassicus* in the same scale as *G. triassicus* to show the size ratio. (**B**) Alternative scenarios proposed for the Archaeognatha (Machiloidea and *Dasyleptus*) evolution; left: evolution of modern-type archaeognathans in Permian-Triassic Period from a *Dasyleptus*-like ancestor; right: evolution of modern-type archaeognathans in Silurian Period. Horizontal bars on branches represent the fossil record: in black those of sure attribution to Archaeognatha, in grey the Devonian specimens. KSZ: Kalkschieferzone; *: the most recent common ancestor (MRCA) of insect is dated according to Misof *et al*.^47^, whereas the MRCA of †Monura and extant lineages of Archaeognatha is placed before the fossil from Gaspé Peninsula (Early Devonian)^16^; dashed vertical line of the dendrogram is reported when no information on the date of the cladogenetic event is available.

The discovery of *G. triassicus*, a representative of Machilidae, besides tracing the origin of this lineage back to the Middle Triassic and extending the range of this group by approximately 200 My, sheds light also on the evolution of archaeognathan body organization. Archaeognatha with a different body organization co-occur in the same stratigraphic unit at Monte San Giorgio: (*i) G. triassicus* representing the new lineage with the presence of well developed cerci and with filum terminale and a large, possibly arched, metathorax supporting jumping capabilities; and (*ii) D. triassicus,* the more ancestral-type, surviving the end-Permian mass extinction (Fig. 4). The latter, according to the fossil record^28^,^29^, was near to its extinction while the former was just blooming.

It has been observed that representatives of *Dasyleptus* markedly resemble juveniles of extant species of Machiloidea^25^,^36^. Therefore, two hypotheses could be formulated as possible explanations concerning of the co-occurrence of these two forms: (*i*) representatives of *Dasyleptus*, including *D. triassicus*, recovered from Upper Carboniferous to Middle Triassic, represent immature stages of Machiloidea; or, (*ii*) fossils described as *Dasyleptus* are representatives of separate species. Even if the first hypothesis is still debated^25^,^36^,^37^,^38^, Rinehart and colleagues^39^, identified six instars in *Dasyleptus brongniarti* from Kuznetsk Formation (Middle Permian) and estimated an adult length of 15–20 mm (including the filum terminale). The authors establish that most specimens of *Dasyleptus* should represent adults and their morphology would therefore be an ancestral adult condition for archaeognathans. The morphology of modern archaeognathans, including *G. triassicus*, would then be a derived condition (Fig. 4) representing an example in which ontogeny recapitulates phylogeny (seen in juvenile machilids, hence a case of peramorphosis).

The evolutionary scenario we propose differs from that so far accepted (Fig. 4) since it postpones the divergence between Machiloidea and *Dasyleptus*. The time and the drivers for the evolution of the new body organization in this lineage of apterous insects are currently unknown. Possible causes could include peculiar paleoenvironmental conditions at the end of the Permian and in the Early Triassic. The high temperatures at the P/T boundary and during the Smithian^40^ may have favored the small size of *Dasyleptus*. Conversely, the switch to a cooler climate during the Spathian^40^ in association with the adaptive advantage provided by body size diversification during the biotic recovery following the end-Permian mass extinction^41^ could be considered the propulsive forces that led to the evolution of giant bristletails with new body organization and jumping capability. An alternative hypothesis relies on a possible, not yet identified abiotic event or on a series of events having occurred during the Middle Triassic that significantly contributed to the renewal of insect lineages. The last hypothesis is supported also by the high rates of insect lineage turnover, origination and extinction in the Middle Triassic^42^,^43^, where some Paleozoic insect lineages become extinct and others have their first occurrence in the same periods. Our findings, associated with the presence of *D. triassicus* on the same stratigraphic unit, support this interpretation of insect evolution. However, the evolution of *G. triassicus* during the Permian, or in earlier period, cannot be ruled out on the basis of currently available data. Studies integrating further fossil evidences and molecular data are required to shed light in the evolution of extant representatives of Machilidae.

## Materials and Methods

### Specimens collection

The two specimens used in this study were collected during the fieldwork activities carried out between 1997 and 2003 in the Lower Kalkschieferzone (KSZ), the uppermost part of the Meride Limestone, at the Val Mara site D near Meride, on the Swiss side of UNESCO World Heritage site of Monte San Giorgio (Italy-Switzerland). Specimens belonging to *Machilis* sp. were collected in Baggero (CO – Italy) in order to isolate the ventral nerve chord and perform the comparison with that of the fossil *G. triassicus*.

### Image acquisition

Direct observations and measurements were performed using a stereomicroscope Leica MS5 with an ocular micrometer. The specimens were photographed under two different settings. First, macrophotography was performed under cross-polarized light with a Canon Rebel T3i with a MP-E 65 mm lens and a Canon Macro Twin Flash MT 24EX, taking several image stacks of adjacent areas to achieve an entirely sharp high-resolution image. The stacks were subsequently fused and stitched with Combine ZM/ZP or Image Analyzer and Adobe Photoshop CS3. Additionally, microphotography using autofluorescence was taken out with a Keyence BZ-9000, again recording image stacks processed in the same way. The autofluorescence of the specimens enhances the contrast against the matrix^44^,^45^.

To isolate the ventral nerve chord, dissections of *Machilis* sp were performed under the stereomicroscope Zeiss Axio Zoom V16 and images of ganglia were acquired with the digital camera Zeiss Axiocam 506.

## Additional Information

**How to cite this article**: Montagna, M. *et al*. Central nervous system and muscular bundles preserved in a 240 million year old giant bristletail (Archaeognatha, Machilidae). *Sci. Rep.* **7**, 46016; doi: 10.1038/srep46016 (2017).

**Publisher's note:** Springer Nature remains neutral with regard to jurisdictional claims in published maps and institutional affiliations.

## Supplementary Material

Supplementary Information
