# Supplementary material for: Central nervous system and muscular bundles preserved in a 240 million year old giant bristletail (Archaeognatha: Machilidae)
Source: Sci Rep. 2017 Apr 7;7:46016. doi: 10.1038/srep46016 (PMC5384076; doi:10.1038/srep46016)

Supplementary Information for

Central nervous system and muscular bundles preserved in 240 million year old giant bristletail (Archaeognatha, Machilidae)

Matteo Montagna*, Joachim T. Haug, Laura Strada, Carolin Haug, Markus Felber, Andrea Tintori

*correspondence to: matteo.montagna@unimi.it

This file includes:

Supporting Notes 1 to 3

References

Supplementary Figure 1

Supplementary Note 1. Geology and Stratigraphy of Middle Triassic succession at Monte San Giorgio.

1.1 Geology

Monte San Giorgio (split by the boundary across Italy and Switzerland) is one of the most renowned among the Middle Triassic sites in the world, since it concentrates several marine vertebrate levels in a small area of approximately 20 square km (45). The Swiss side of Monte San Giorgio has been inscribed in the UNESCO World Heritage List in 2003, joined by the Italian side in 2010, for the global significance of its fossil marine fauna.

The Monte San Giorgio basin is located at the western termination of the South-Alpine domain situated on a passive continental margin open to the tropical western Neo-Tethys (46), which was progressively submerged by a long-term transgression from the east. Its location resulted in a peculiar sedimentary succession, showing the onsetting, at least temporarily, of severe dysoxic to anoxic bottom water conditions (33, 47). The marine ingression reached the eastern South-Alpine domain in the Late Permian and the westernmost (i.e. west of Lake Como) South-Alpine domain in the Late Anisian times. The intensive Middle Triassic tectonics made the palaeogeographic scenario more complex, resulting in a structural compartmentalization of the area (48).

The east–west extension of the Monte San Giorgio basin is estimated to have been about 10 km or up to 20 km if it was located in the same basin as the Perledo–Varenna Formation outcropping to the east of Lake Como (49-50). Basin depths in MSG are regarded as varying between 30 and 130 m and 160–260 m for the Perledo–Varenna Formation (47, 48, 51-53).

1.2 Stratigraphy

The Triassic succession at Monte San Giorgio spans from the Olenekian-Middle Anisian to the Norian, possibly the Rhaetian beds being eroded at the end of the Triassic - beginning of Jurassic. The sequence starts with fluvio-deltaic deposits dated possibly to the Lower Triassic Servino Formation and surely to the Middle Anisian Bellano Formation, unconformably overlying a Lower Permian volcanic basement (54).

The Bellano Formation Middle Anisian sediments testify the progressive transgression of a shallow sea from the east and the initiation of carbonate platform growth (San Salvatore Dolomite/Esino Limestone). Dolomitized microbial limestones, characterized by stromatolitic lamination, were deposited in a shallow subtidal to intertidal environment (Lower Salvatore Dolomite). The overlying Besano Formation, San Giorgio Dolomite and Meride Limestone, forming an approximately 600 m thick sequence, were deposited from the Late Anisian through most of the Ladinian during the formation of an intraplatform basin with restricted circulation (47, 50, 55).

The Kalkschieferzone (KSZ) is the uppermost part of the Meride Limestone. It forms a 120 m thick level of thin-bedded, mostly laminated, limestones and marlstones. It represents the latest stage of the intraplatform basin, recording strong seasonal variations of precipitations leading to sudden changes in salinity (32), which was progressively buried by an increasing input of siliciclastic material from the nearby small islands and large emerged land (56).

The KSZ has been recently dated (54) to 239.51 ± 0.15 Ma, somewhat older than previously thought.

Besides providing new radionuclide dating for the Meride Limestone, Stockar (54) also highlighted how sedimentation rates in that area can be estimated around 180-200 m/Ma, much higher than in surrounding areas (estimated 8 m/Ma for the Buchenstein facies of the Bagolino section in the Brescia area).

The depositional environment of the KSZ was that of a shallow lagoon, adjacent to a carbonate platform (S. Salvatore Dolomite). Toward East-Northeast it faced a deeper basin (Perledo-Varenna Formation) and the complex system of carbonate platforms of the Esino Formation further to the East (Grigna Mountain), with somewhat limited connection to the open and deeper sea (32, 33, 56). Sedimentation took place below wave base and with an often anoxic bottom, as indicated by common laminated limestone or marly-limestone layers and the almost general absence of bioturbation (32, 33, 56, 57). Quite common are also clay-chips beds, often rich also in dark algal-film fragments, probably related to storms affecting the shallower part of the basin or the threshold toward the open waters (32, 33).

During the deposition of the uppermost Meride Limestone (the Kalkschieferzone Member), the fresh water influence became stronger and stronger: conchostracans and insects point to a quite close land with superficial fresh-water ponds, permanent or seasonal, as suggested by the number of conchostracan-rich surfaces (32, 58).

Supplementary Note 2. The fossil assemblages and the paleoenvironment in the Late Ladinian of Monte San Giorgio

2.1 General information

During about 25 years of excavations in the Lower and Middle KSZ, a number of species have been reported from this upper Member of the Calcare di Meride. However, the general biodiversity of this Member is quite low, as apart from about 20 fish species actually subdivided in at least two different assemblages (32, 56, 59-63), among the macroremains, apart from the insects, we recorded only the nothosaurid *Lariosaurus valceresii* (57, 64), possibly three crustacean taxa (the mysidiacean *Schimperella* sp. n., the conchostracan *Laxitextella* sp. n. (58) and one very rare decapods) and a few terrestrial plant remains. On the other hand, if we compare the fossil assemblages in the KSZ with those from the lower Calcare di Meride Cava Inferiore, Cava Superiore and Cassina, we do not see many differences in the number of marine vertebrate and invertebrate species found in each single level (65; 66). Thus, the only major difference is related to the presence of the insects and fresh-water conchostracans in the KSZ.

Actually, during the deposition of the Calcare di Meride, the fresh water influence became stronger and stronger and in the KSZ no sure stenohaline organism has been found, leaving apart the nothosaurid *Lariosaurus* and most of the fishes. In fact, many fish genera have been found also in other localities that can be considered surely marine, such as Luoping, in southern China (67, 68) (and A.T. pers. obs.) or Perledo along the eastern coast of the Lario Lake (56, 67) or just the Besano-Formation in this same Monte San Giorgio area (69).

Conchostracans and insects point to a quite close land with superficial fresh-water ponds, permanent or seasonal, as suggested by the number of conchostracan-rich surfaces. Tintori (32) and Tintori and Brambilla (56) proposed an alternation between dry and very

rainy season, a monsoonal-like climate where heavy rains could suddenly affect the KSZ salted basin causing mass mortality events in the marine fauna, mainly fishes (32, 33). A further support to the fresh water causing mass mortality in a marine basin after flooding the nearby land is given by the assemblage yielding *Dasyleptus triassicus* even if Bechly and Stockar (28) did not interpret correctly what was the taphonomic history of the surface yielding the three specimens of *D. triassicus*. It is clear that having a surface of only two square meters yielding three specimens of this terrestrial insect together with ‘diffuse small land plant remains’ and five fish specimens (28), this must be considered as a mass mortality one, owing to the flooding that brought the insects and the plant remains in the basin and also killed the small fishes. Other than these uncommon peculiar surfaces, the number of fish specimens by square meter is actually very low (32, 33). Actually, Bechly and Stockar (28) did not find any other fossil in the excavation site yielding *D. triassicus* and the small fishes, even if the investigated sequence is over two meters thick, proving that the fossiliferous surface in the whole is strictly related to a flooding that caused also the death of marine fishes. Thus, it is evident that we can consider a mass mortality surface in the KSZ when we have just only one specimen per square meter of a single surface, especially if all are about the same size and they belong mostly to a single species.

As already pointed out (32, 33, 56), the major mortality of marine dwellers was concentrated possibly in a single season of the year, possibly the rainy one. Stormy heavy rains could help in bringing insects to the basin from the nearby-emerged land by both running waters and winds.

2.2 The insect assemblages

During the fieldwork carried out between 1997 and 2003 in the Lower KSZ at the Val Mara site D near Meride, on the Swiss side of Monte San Giorgio, a remarkably diverse assemblage of 19 insect specimens were collected. They include whole individuals and fragments, adult specimens and larval stages. Some of the specimens have been described to the genus or species level, namely: the ephemeropteran (mayfly) (70) *Tintorina meridensis* Krzeminski and Lombardo 2001, two coleopterans (beetles) (13, 70) (*Praedodromeus sangiorgensis*, Strada et al., 2014; *Notocupes* sp., Krzeminski and Lombardo 2001); the archaeognathans (28) *Dasyleptus triassicus* Bechly and Stockar 2011 and *Gigamachilis triassicus* (this paper). The remaining specimens are still under study to confirm their assignment to eight different linages ("orders") (13). Noteworthy, the entomofauna of Monte San Giorgio includes terrestrial groups, with both phytophagous and predatory habits, and aquatic groups, collected both as larvae (?Plecoptera) and as adults (Ephemeroptera, ?Coptoclavidae). Coleoptera are the most common group with six specimens, both whole individuals and fragments.

Supplementary Note 3. Description of paratype specimen MCSN8466

Specimen will be deposited at Museo Cantonale di Storia Naturale di Lugano (MCSN) – Switzerland.

Habitus (Supplementary Fig. S1 online). Total length ~27.7 mm. Preserved only the thorax (meso- and meta-) and the first six abdominal metameres, specimen visible in ventral view.

Thorax. Light impressions of meso- and metathorax preserved. Structures attributable to

metatrochanters visible.

Abdomen. Abdominal metameres from I to VI visible, coxopodites I and II well preserved, bearing eversible vesicles and styli on the right side. A styli-like appendage presents on the right side of metamere V.

References

45. Tintori, A. A new species of *Saurichthys* (Actinopterygii) from the Middle Triassic (Early Ladinian) of the Northern Grigna Mountain. *Riv. Ital. Paleontol. S.* **119**, 387–302 (2013).
46. Stampfli, G. M. & Borel, G. D. A plate tectonic model for the Paleozoic and Mesozoic constrained by dynamic plate boundaries and restored synthetic oceanic isochrons. *Earth Planet Sci. Lett.* **196**, 17–33 (2002).
47. Tintori, A. Fish taphonomy and Triassic anoxic basins from the Alps: a case history. *Riv. Ital. Paleontol. S.* **97**, 393–408 (1992).
48. Gaetani, M., Gnaccolini, M., Jadoul, F. & Garzanti, E. Multiorder sequence stratigraphy in the Triassic system of the Western Southern Alps. Mesozoic and Cenozoic Sequence Stratigraphy of European Basins. *SEPM Spec. P* **60**, 701–717 (1998).
49. Gianotti, R. & Tannoia, G. Elementi per una revisione stratigrafico-paleontologica del Trias medio superiore della regione compresa tra il Lario e il Ceresio. *Atti Ticinensi Sci. Terra* **31**, 434–445 (1988).
50. Bernasconi, S. M. in *Geochemical and microbial controls on dolomite formation in anoxic environments: A case study from the Middle Triassic (Ticino, Switzerland)*

(eds Füchtbauer, H., Lisitzyn, A. P., Milliman, J. D. & Seibold, E.) *Contributions to Sedimentology* Vol. 19, 1–109 (1994).

51. Senn. A. Beiträge zur Geologie des Alpensüdrandes zwischen Mendrisio und Varese. *Eclogae Geol. Helv.* **18**, 552–632 (1924).

52. Gaetani, M. et. al. L. An anoxic intraplatform basin in the Middle Triassic of Lombardy (Southern Alps, Italy): Anatomy of a hydrocarbon source. *Riv. Ital. Paleontol. S.* **97**, 329–354 (1992).

53. Sciunnach, D., Gaetani, M. & Roghi, G. La successione terrigena pre-Ladinica tra Lugano e Varese (Canton Ticino, Svizzera; Lombardia, Italia). *Geol. Insubrica* **11**, 45–61 (2015).

54. Stockar R., Baumgartner P.O. & Condon D. Integrated Ladinian bio-chronostratigraphy and geochrononology of Monte San Giorgio (Southern Alps, Switzerland). *Swiss J. Geosci.* **60**, 239–269 (2012).

55. Furrer, H. The Kalkschieferzone (Upper Meride Limestone; Ladinian) near Meride (Canton Ticino, Southern Switzerland) and the evolution of a Middle Triassic intraplatform basin. *Eclogae Geol. Helv.* **88**, 827–852 (1995).

56. Tintori A. & Lombardo C. Late Ladinian fish faunas from Lombardy (N-Italy): stratigraphy and paleobiology. In *Proceedings of the Symposium "Mesozoic fishes: systematics and fossil record"* (eds Arratia, G. & Schultze, H. P.) 495–504 (Verlag F. Pfeil, 1999).

57. Tintori A. & Renesto S. A new *Lariosaurus* from the Kalkschieferzone (Uppermost Ladinian) of Valceresio (Varese-N. Italy). *Boll. Soc. Paleontol. I.* **29**, 309–319 (1990).

58. Tintori, A. & Brambilla, E. Sexual dimorphism in a Conchostracan population from the Late Ladinian of Southern Calcareous Alps (N. Italy). *Contr. Paleontol. Mus. Univ. Oslo* **364**, 65–66 (1991).

59. Lombardo, C. Sexual dimorphism in a new species of the actinopterygian *Peltopleurus* from the Triassic of Northern Italy. *Palaeontology* **42**, 741–760 (1999).

60. Lombardo, C. Actinopterygians from the Middle Triassic of Northern Italy and Canton Ticino (Switzerland): anatomical descriptions and nomenclatural problems. *Riv. Ital. Paleontol. S.* **107**, 345–369 (2001).

61. Lombardo, C. *Coelatichthys* gen. n.: a new palaeonosciform from the Middle Triassic of Northern Italy and Canton Ticino (CH). *Riv. Ital. Paleontol. S.* **108**, 399–414 (2002).

62. Lombardo C. & Tintori A. New perleidiforms from the Triassic of the Southern Alps and the revision of Serrolepis from the Triassic of Württemberg (Germany). In *Proceedings of the Symposium "Mesozoic Fishes 3 - Systematics, Paleoenvironments and Biodiversity"* (eds Tintori A. & Arratia G.) 179–196 (Verlag F. Pfeil, 2004).

63. Tintori A. & Lombardo C. A new early Semionotidae (Semionotiformes, Actinopterygii) from the Upper Ladinian of Monte San Giorgio area (Southern Switzerland and Northern Italy). *Riv. Ital. Paleontol. S.* **113**, 369–381 (2007).

64. Renesto S., Lombardo C., Tintori A. & Danini G. Nothosaurid embryos from the Middle Triassic of Northern Italy: an insight into the viviparity of Nothosaurs? *J. Vertebr. Paleontol.* **23**, 958–961 (2003).

65. Bürgin, T. Pesci fossili del Triassico Medio di Monte San Giorgio (Svizzera meridionale) e della zona di Besano (Italia settentrionale). *Geol. Insubrica* **3**, 1–9 (1998).
66. Lombardo C., Sun Z.Y., Tintori A., Jiang D.Y. & Hao W.C. A new species of the genus *Perleidus* (Actinopterygii: Perleidiformes) from the Middle Triassic of Southern China. *Boll. Soc. Paleontol. I.* **50**, 75–83 (2011).
67. Lombardo C., Rusconi M. & Tintori A. New perleidiform from the Lower Ladinian (Middle Triassic) of the Northern Grigna (LC). *Riv. Ital. Paleontol. S.* **114**, 263-272 (2008).
68. López-Arbarello A. et al. New species of *Sangiorgioichthys* Tintori and Lombardo, 2007 (Neopterygii, Semionotiformes) from the Anisian of Luoping (Yunnan Province, South China). *Zootaxa* **2749**, 25–39 (2011).
69. Bürgin T. Middle Triassic marine fish faunas from Switzerland. In *Proceedings of the Symposium "Mesozoic fishes: systematics and fossil record"* (eds Arratia, G. & Schultze, H. P.) 481–494 (Verlag F. Pfeil, 1999).
70. Krzeminski W. & Lombardo C. New fossil Ephemeroptera and Coleoptera from the Ladinian (Middle triassic) of Canton Ticino (Switzerland). *Riv. Ital. Paleontol. S.* **107**, 69–78 (2001).

Supplementary Figures

Supplementary Figure 1. *Gigamachilis triassicus* paratype. (A) Overview. **(B)** Colour-marked version of **A**; remains of thoracopods in red; coxae and coxopodites in yellow;

derivatives of endopod in green; derivatives of exopod in blue. Abbreviations: a3? = possible third abdominal segment; a5? = possible fifth abdominal segment; ap1? = possible first abdominal appendage; ap2? = possible second abdominal appendage; rtp? = possible remains of anterior thoracopods; tp3? = possible third thoracopod.

Figure Supplementary 1.

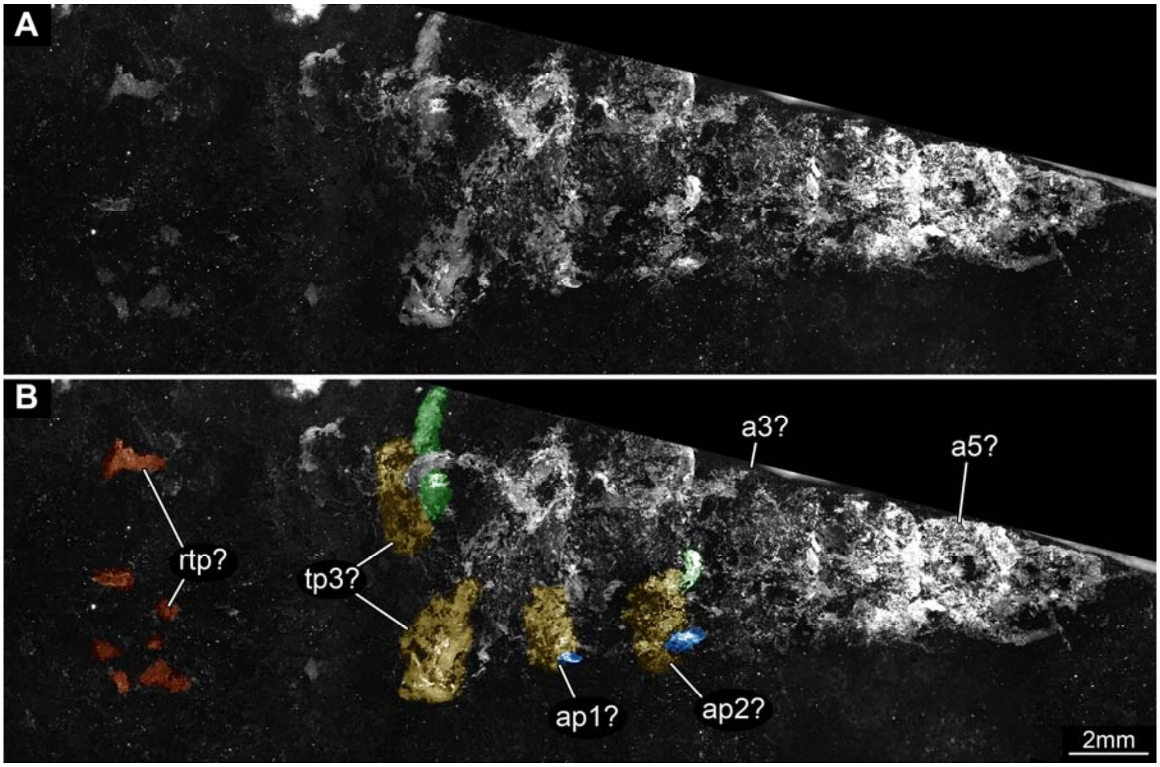

Supplement: Supplementary Information [file srep46016-s1.pdf]
